# A retrospective analysis of mission reports in the national Swedish Police Registry on mountain rescue 2018–2022: here be snowmobiles

**DOI:** 10.1186/s13049-024-01210-4

**Published:** 2024-04-25

**Authors:** Anton Westman, Johanna Björnstig

**Affiliations:** 1 Centre for Disaster Medicine, Department of Diagnostics and Intervention, Umeå University, Umeå, 901 87 Sweden; 2 Department of Anaesthesia and Intensive Care Medicine, Karolinska University Hospital, Huddinge, Stockholm, Sweden

**Keywords:** Wilderness medicine, Mountaineering, Rescue work, Emergency medical services

## Abstract

**Background:**

Increasing mountain activity and decreasing participant preparedness, as well as climate change, suggest needs to tailor mountain rescue. In Sweden, previous medical research of these services are lacking. The aim of the study is to describe Swedish mountain rescue missions as a basis for future studies, public education, resource allocation, and rescuer training.

**Methods:**

Retrospective analysis of all mission reports in the national Swedish Police Registry on Mountain Rescue 2018–2022 (*n* = 1543). Outcome measures were frequencies and characteristics of missions, casualties, fatalities, traumatic injuries, medical conditions, and incident mechanisms.

**Results:**

Jämtland county had the highest proportion of missions (38%), followed by Norrbotten county (36%). 2% of missions involved ≥ 4 casualties, and 44% involved ≥ 4 mountain rescuers. Helicopter use was recorded in 59% of missions. Non-Swedish citizens were rescued in 12% of missions. 37% of casualties were females. 14% of casualties were ≥ 66 or ≤ 12 years of age. Of a total 39 fatalities, cardiac event (*n* = 14) was the most frequent cause of death, followed by trauma (*n* = 10) and drowning (*n* = 8). There was one avalanche fatality. 8 fatalities were related to snowmobiling, and of the total 1543 missions, 309 (20%) were addressing snowmobiling incidents. Of non-fatal casualties, 431 involved a medical condition, of which 90 (21%) suffered hypothermia and 73 (17%) cardiovascular illness.

**Conclusions:**

These baseline data suggest snowmobiling, cardiac events, drownings, multi-casualty incidents, and backcountry internal medicine merit future study and intervention.

## Background

The International Commission for Alpine Rescue (ICAR) assesses that mountain tourism is attracting larger numbers of unprepared adepts, leading to a rise in accidents [1]. In Italy, Canada and Scotland, the number of mountain search and rescue (SAR) operations has grown [2–4], and in French mountain SAR, more than 10% are rescued in an event involving ≥ 4 victims [5]. Males and elderly persons have been described as having an elevated risk, and predominant causes of death are trauma, hypothermia, cardiac death, and avalanches [6–8]. A national park in Taiwan has a fatality incidence per 100 000 visitors of 0.24 [9], and the UK suffered 550 fatal incidents in 6814 mountain rescue missions 2002–2006 [10]. Climate change must also be factored in, as in the 2022 Marmolada glacier collapse in Italy, with eleven fatalities and SAR hampered by thunderstorms [11]. 

The Scandes mountain range forms the border between Norway and Sweden, stretches into Finland, and is a core region of Sápmi, the traditional land of the Indigenous Sámi [12]. Human activities in the Scandes include reindeer-herding, mining, power production, forestry, hunting and fishing, and tourism. In medical literature related to these activities, a substantial body of studies exists for Norwegian mountain rescue, but few previous studies have addressed Swedish mountain incidents. Yet, in 2022, 88% of major Swedish mountain tourism executives stated an increase in inexperienced visitors, and 60% a decrease in visitor risk awareness [13]. In view of worrying trends internationally, a need for study of mountain emergencies also on the Swedish side is indicated, where some Norwegian results may not be fully applicable due to differences in e.g. methods [14] and topography – even though anecdotal information suggests frequent assistance in Sweden by Norwegian mountain SAR helicopter services. Mountain SAR in Sweden is the responsibility of its police, aided by the Swedish Mountain Rescue volunteer corps, trained and equipped by the police and working under a police rescue leader [15]. Every mountain rescue mission generates a report saved in a police registry, providing an opportunity for explorative research. The aim of the current study is to describe Swedish mountain rescue police registry data, as a baseline for future studies, public education, resource allocation, and rescuer training.

## Methods

### Research design

Descriptive register study.

### Outcome measurements

The primary outcome measurements were frequencies and characteristics of missions. Secondary outcome measurements were number and demographic characteristics of casualties; frequencies of traumatic injuries, medical conditions, and fatalities; and incident mechanisms. No prespecified hypotheses were stated.

### Subjects

All data recorded in the Swedish Police Registry on Mountain Rescue (Swedish: *Polisens Fjällräddnings Uppdragsstatistik*) 2018–2022 were extracted, yielding mountain rescue mission statistics from the four counties (geographic subdivisions of Sweden) through which the Swedish Scandes mainly run: Norrbotten, Västerbotten, Jämtland, and Dalarna. The time period from 2018 and onwards was chosen in dialogue with the Police, since the data recording was methodologically improved from that year. We investigated data from previous years (1999–2017) and could confirm marked quality improvements from 2018, e.g. comprehensive data from all four counties. Prior to 2018, we found possible but unconfirmed cases in available raw data (*n* = 1619: Dalarna 210 cases, Jämtland 728 cases, Norrbotten 565 cases, Västerbotten 116 cases) lacking an individual report with a police reference number, and containing no information. All of these possible but unconfirmed cases were between the years 2000–2006. The year 2007 had no reports recorded in raw data made available to us. The study was approved in advance by the Swedish Ethical Review Authority (dnr 2022-03164-01).

### Data management and statistics

Data were extracted in electronic spreadsheet format per year and county, each mission report designated by a police reference number, and a database for analysis was created in which raw data was preserved. No imputations were applied (four reports stating ”group of people” were conservatively assessed as three persons per group) and no inferential statistics were calculated. The binary sex categorization by the police was used since no gender (socially constructed roles) data were available to us. The STROBE (Strengthening the Reporting of Observational Studies in Epidemiology) checklist was used as far as applicable [16]. 

## Results

### Mission characteristics

A total 1543 recorded missions 2018–2022 were distributed as follows: Jämtland 586 missions (38%), Norrbotten 553 missions (36%), Dalarna 220 missions (14%), Västerbotten 184 missions (12%). 1229 missions (80%) were recorded as rescue, and 314 (20%) as search missions. Helicopter use was recorded for 913 (59%) missions, of which 113 (7% of total) involved multiple helicopters and 800 (52%) a singular helicopter. Types of helicopter were distributed as follows: 434 (28%) ambulance helicopter, 313 (20%) police helicopter, 225 (15%) civilian helicopter, 51 (3%) Norwegian helicopter (of any type), and 16 (1%) Swedish maritime SAR helicopter. Of all missions, 771 (50%) were carried out November through April. The most afflicted month was August with 268 (17%) missions and the least afflicted month was November with 7 (0.5%) missions. Time of day was found to be recorded in only 234 (15%) cases, and was not analysed. A total 37 missions (2%) involved ≥ 4 casualties, and a total 682 missions (44%) involved ≥ 4 mountain rescuers. Non-Swedish citizens were rescued in 181 (12%) missions, the most frequent foreign nationalities being Norwegian (51 missions; 3%) and German (28 missions; 2%). A total 23 foreign nationalities were found among the rescued.

### Casualties and fatalities

A total 1857 persons could be confirmed as found dead or alive, with sex recorded for 1476 casualties; 923 males (63%) and 553 females (37%). Age was reported for 1484 persons of which 51 (3%) were ≤ 12 years of age, and 160 (11%) were ≥ 66 years of age. A total 39 fatalities were recorded, with cardiac event (14 persons; 36% of fatalities) recorded as the most frequent cause of death (Table 1). All 8 drownings (21% of fatalities) occurred March-July; one was a skiing and one was a snowmobiling incident, and the other six where categorized as hiking, boating or canoeing.

**Table 1:** Stated causes of death for fatalities in the Swedish Police Registry on Mountain Rescue 2018–2022 (*n* = 39)

Cause of death	Number of fatalities	% of fatalities
Cardiac event	14	36
Trauma	10	26
Drowning	8	21
Avalanche	1	3
Suicide	1	3
Sudden infant death syndrome	1	3
Not stated	4	10
**TOTAL**:	**39**	**100**

### Non-fatal medical conditions and traumatic injuries

Of non-fatal casualties, 431 involved a medical condition, with the seven most common ranked by frequency as follows: Hypothermia (90 persons; 21%), Cardiovascular (73 persons; 17%), Exhaustion (68 persons; 16%), Neurologic (60 persons; 14%), Gastrointestinal (56 persons; 13%), Respiratory (16 persons; 4%), Endocrine (13 persons; 3%). Several of these had a concurrent trauma registered, e.g. hypothermia and lower limb injury. Anatomical location of traumatic injuries could be identified for 771 injuries (Table 2). A total 6 non-fatal casualties were recorded as burn injuries, and 4 as gunshot wounds.

**Table 2:** Anatomical location of non-fatal traumatic injuries recorded in the Swedish Police Registry on Mountain Rescue 2018–2022 (*n* = 771)

Anatomical location	Number of injuries
Lower limb	425
Upper limb	139
Head/Neck	113
Back	67
Chest/Abdomen/Pelvis	27
**Total**:	**771**

### Incident mechanisms

Of the total 1543 missions, 737 (48%) addressed incidents related to hiking; 393 (25%) to skiing; and 309 (20%) to snowmobiling. Of a total 39 fatalities, 8 (21%) were related to snowmobiling. 48 (3% of total number) missions were avalanche SAR, including proactive searches resulting in no persons found or missing. Of these 48 avalanches, 28 (58%) were related to skiing and 5 (10%) to snowmobiling, and 33 (69%) occurred in Jämtland. One avalanche victim died. Free-text data indicated that a majority of the 8 drownings were in ice-free conditions.

## Discussion

The two main findings of this study are the high frequencies of snowmobile incidents and of drownings. While no prespecified hypotheses were stated for this explorative baseline study, the Swedish results were expected to be more similar to international predecessors than they turned out to be. In e.g. Japan, cardiac mountain deaths are not as prominent as in our material and drownings constitute only 3.3% of Japanese mountain fatalities [8]. The proportion of females was higher (37% of casualties with recorded sex) than for many international materials; the corresponding number for France is 17% females [5]. Even though our comparatively small sample size must be considered, it cannot be ruled out that Sweden has outlier tendencies, to be further explored.

One such outlier tendency could be the enormous popularity of snowmobiling in Sweden. Its > 150 snowmobile clubs maintain 28 000 km of snowmobile trails, equalling 70% of the Earth’s circumference [17]. A recent survey found that 70% of Swedish snowmobile riders who drive in avalanche terrain do so without avalanche equipment [18]. In Utah, snowmobiling accounted for 47% of avalanche fatalities 2006–2018 [19], and given the survey result indicating avalanche risk negligence among Swedish snowmobilers, a substantial number of snowmobiling avalanche fatalities could have been expected in Sweden, but this was not the case. Again, our comparatively small sample size must be considered. 10% of all avalanche cases were related to snowmobiling. Finding that snowmobiling generates 20% of the need for rescue in the ”pristine” Swedish Scandes, we asked the Mountain Safety Council of Sweden for comment, and were suggested that there may in fact be an even higher frequency of snowmobile incidents, since some may be classified as traffic and not recorded in the mountain rescue registry. Systematic study of comprehensive current data for snowmobile incidents in Sweden is suggested, but a call for action may be warranted already from these baseline results. Setting higher standards for driver training and certification could aid in reducing risks [20], and discussions with the industry may be of value, perhaps tied to the electric transition: A *smart snowmobile* could possibly warn its rider before an incident, and call for help (including transceiver technologies) if there is one.

Even though substantially more people drown in Swedish mountains than die in avalanches (the herein described ratio is 8 − 1), the Swedish Mountain Rescue volunteer corps are not trained or equipped for water rescue. Knowledge transfer from its maritime volunteer corps equivalent, the Swedish Sea Rescue Society, could be of value. An in-depth study of autopsies and records from pre- and intrahospital sources is suggested to characterize mechanisms and provide data for improvements in prevention and rescue. The Swedish Scandes contain a superfluity of water bodies that climate change may alter seasonal characteristics of, possibly jeopardizing e.g. trail bridges. Off-piste skiers are asked to train and equip (beacon, shovel, probe, airbag) themselves for avalanche - a corresponding drowning risk preparedness could be asked of certain mountain visitor subpopulations.

It must be noted that in the present material, 36% of fatalities were a cardiac event. The substantial cardiovascular proportion in the nontraumatic illness distribution appears to validate that a high proportion of fatalities in Swedish mountains are cardiac events. From the police data, we could not satisfactorily determine explanatory risk factors, but suggest cardiovascular incidents for prioritized future study, not at least since several well-known and well-studied interventions might be transferable to this environment, such as increased bystander cardiopulmonary resuscitation skills and increased availability of (e.g. drone-delivered) automated defibrillators. Future training and equipping of mountain rescue volunteers may benefit from an increased cardiovascular preparedness.

In its guidelines for mountain multi-casualty incidents (MCIs), ICAR notes that, compared with an urban environment, a lower number of casualties may be considered an MCI because resources are more limited [21]. What number of victims would overwhelm Swedish mountain SAR, pushing a mountain incident out of control? If the answer is ≥ 4 casualties, then there was a potential for 37 MCIs 2018–2022, i.e. seven per year. It seems advisable to explore disaster preparedness in this context, especially in view of there being evidence-based guidelines.

The fact that 14% of casualties were ≥ 66 or ≤ 12 years of age raises questions about vulnerability, and factoring in a postulated general decrease in preparedness for the mountain environment is worrying. There was one case of sudden infant death syndrome. The heterogeneity of victim nationalities raises questions about preparedness for Scandinavian conditions [22]. 

The frequency (90 cases) of hypothermia is unreliable since it is based on medical layperson descriptions, lacking objective data on body temperature. None of the fatalities had hypothermia as stated cause of death, whereas in Japan 14.7% of mountain fatalities died due to hypothermia [8]. Does Sweden have unexpectedly low incidence or poor diagnostics? As for the related (possibly overlapping) issue of drownings, an in-depth study of autopsies and records from pre- and intrahospital sources is suggested. Yet, already herein described cases of hypothermia, if seen in view of a general decrease in preparedness combined with a high proportion of vulnerable populations, may warrant public education measures as well as resource planning [23]. In the anatomical distribution of non-fatal injuries, the injuries to the head-neck-complex may merit particular future investigation, having prevention (armour) and rescue (stabilization) implications.

## Limitations

Limitations of this study include unknown validity and reliability of the registry used and a lack of medical records.

## Conclusions

These baseline data suggest snowmobiling incidents, cardiac events, drownings, multi-casualty incidents, and backcountry internal medicine for future study; the results for snowmobiling and drownings are perhaps already strong enough to suggest action. Experiences of rescuers and survivors are implicated for future study from our findings of complex cases in challenging environments. Future climate instability may cause incident mechanisms and conditions for medical evacuation to deviate from herein described baselines, suggesting need for proactive study and intervention.

## Data Availability

Data (in Swedish) is available from the corresponding author upon reasonable request.

## Abbreviations

ICAR: International Commission for Alpine Rescue
SAR: Search and Rescue
STROBE: Strengthening the Reporting of Observational Studies in Epidemiology
MCI: Multi-Casualty Incident

## References

[1] Consensus Guidelines on Mountain Emergency Medicine and Risk Reduction. · ICAR – International Commission for Alpine Rescue [Internet]. [cited 2023 Sep 18]. https://www.alpine-rescue.org/articles/79--consensus-guidelines-on-mountain-emergency-medicine-and-risk-reduction.

[2] Scottish Mountain Rescue reports extremely busy year. BBC News [Internet]. 2023 May 12 [cited 2023 Sep 18]; https://www.bbc.com/news/uk-scotland-highlands-islands-65570949.

[3] Curran-Sills GM, Karahalios A (2015). Epidemiological trends in search and rescue incidents documented by the Alpine Club of Canada from 1970 to 2005. Wilderness Environ Med.

[4] Milani M, Roveri G, Falla M, Dal Cappello T, Strapazzon G (2023). Occupational Accidents among Search and Rescue Providers during Mountain Rescue Operations and training events. Ann Emerg Med.

[5] Vanpoulle M, Lefevre B, Soule B (2021). Mountaineering incidents in France: analysis of search and rescue interventions on a 10-year period (from 2008 to 2018). J Mt Sci.

[6] Gasser B (2022). Deathly accidents while High-Altitude Mountaineering in the Swiss Alps—An observational analysis from 2009 to 2021. Int J Environ Res Public Health.

[7] Amarowicz J, Kumorek A, Boczon K (2019). Age and sex are strongly correlated to the rate and type of Mountain injuries requiring search and rescue missions. Wilderness Environ Med.

[8] Oshiro K, Murakami T (2022). Causes of death and characteristics of non-survivors rescued during recreational mountain activities in Japan between 2011 and 2015: a retrospective analysis. BMJ Open.

[9] Wang SH, Hsu TY, Kuan JT, Chen JC, Kao WF, Chiu TF (2009). Medical problems requiring mountain rescues from 1985 to 2007 in Yu-Shan National Park, Taiwan. High Alt Med Biol.

[10] Mort AJ, Godden DJ (2010). UK mountain rescue casualties: 2002–2006. Emerg Med J EMJ.

[11] Giuffrida A. Italian glacier collapse: rescuers hampered by thunderstorms. The Guardian [Internet]. 2022 Jul 4 [cited 2023 Sep 21]; https://www.theguardian.com/world/2022/jul/04/italy-glacier-collapse-rescue-teams-continue-search-for-missing-hikers.

[12] Hassler S, Sjölander P, Johansson R, Grönberg H, Damber L (2004). Fatal accidents and suicide among reindeer-herding Sami in Sweden. Int J Circumpolar Health.

[13] Sommarfjällenkäten. 2022 - Fjällsäkerhetsrådet [Internet]. [cited 2023 Sep 14]. https://fjallsakerhetsradet.se/om-oss/rapporter/sommarfjallenkaten-2022/.

[14] Mattingsdal H, Abrahamsen HB, Fevang E, Sollid SJM (2022). Static rope Rescue Operations in Western Norway: a retrospective analysis of 141 missions. Wilderness Environ Med.

[15] Fjällräddarna. | Polismyndigheten [Internet]. [cited 2023 Sep 12]. https://polisen.se/om-polisen/polisens-arbete/fjallraddarna/.

[16] Field N, Cohen T, Struelens MJ, Palm D, Cookson B, Glynn JR (2014). Strengthening the reporting of Molecular Epidemiology for Infectious diseases (STROME-ID): an extension of the STROBE statement. Lancet Infect Dis.

[17] 750 000 kr till Sveriges skoterleder! | Svedea [Internet]. [cited 2023 Sep 13]. https://www.svedea.se/press/pressarkiv/750-000-kr-till-sveriges-skoterleder.

[18] Många snöskoterförare kör i lavinterräng utan. lavinutrustning - Nationella Snöskoterrådet [Internet]. [cited 2023 Sep 14]. http://snoskoterradet.se/2023/03/08/pressmeddelande-8-mars-2023-manga-snoskoterforare-kor-i-lavinterrang-utan-lavinutrustning/.

[19] McIntosh SE, Brant-Zawadzki G, Milliner BH, Christensen ED, Nyberg AA, Grissom CK (2019). Cause of death in Utah Avalanche fatalities, 2006–2007 through 2017–2018 Winter Seasons. Wilderness Environ Med.

[20] W JH, Jr WH. On the escape of tigers: an ecologic note. Am J Public Health Nations Health. 1970;60(12):2229–34. 10.2105/ajph.60.12.2229-b PMC1349282 5530409

[21] Blancher M, Albasini F, Elsensohn F, Zafren K, Hölzl N, McLaughlin K (2018). Management of Multi-casualty incidents in Mountain rescue: evidence-based guidelines of the International Commission for Mountain Emergency Medicine (ICAR MEDCOM). High Alt Med Biol.

[22] The Irish Times [Internet]. [cited 2023 Oct 26]. Six Irish people rescued from Swedish mountain. https://www.irishtimes.com/news/ireland/irish-news/six-irish-people-rescued-from-swedish-mountain-1.4653970.

[23] Carlsen AW, Skjaervold NK, Berg NJ, Karlsen Ø, Gunnarson E, Wahba A (2017). Swedish-norwegian co-operation in the treatment of three hypothermia victims: a case report. Scand J Trauma Resusc Emerg Med.

